# Cunningham and the "Daily Express"

**Published:** 1901-01-19

**Authors:** 


### CUNNINGHAM AND THE "DAILY EXPRESS."

The Queen's Bench Division of the High Court of Justice was occupied during three days a week or two ago in the consideration of a case which is one of considerable interest in that, if the verdict had been different from the one arrived at, great discouragement would have been thrown in the way of honest and enterprising journalists in rooting out and exposing abuses dangerous to the public. The case was one in which an impudent attempt was made by an American, who calls himself Cunningham, to obtain damages from our contemporary, the *Daily Express*, for the publication of an article in which the plaintiff and a man named Clark, who was associated with him, were described as a "gang of Yankee sharps," and in which it was said that these "sharps" had hoped to deceive the editor of the *Express* into publishing a glowing account of a fictitious cure for consumption which Cunningham claimed to have discovered. Cunningham said in his evidence that he took an M.D. degree at Vermont in 1890; and as to this he was not cross-examined, although the statements recently published by the Medical Council show that several of the bogus degrees of America are so drawn as to bear a colourable resemblance to those conferred by veritable universities, among which, we believe, the University of Vermont must undoubtedly be numbered. Mr. Lawson Walton, Q.C., in placing the plaintiff's case before the jury, said that the plaintiff had discovered a treatment for consumption by means of subcutaneous injections of stimulating drugs, and that in 1899, assisted by a loan from a friend, he started in practice in consulting-rooms in Oxford Street, and announced his alleged discovery by advertisement in the columns of a London newspaper. Shortly afterwards he "received an intimation from the Medical Defence Society [Union?] that the advertisement was a violation of a rule of the profession. The plaintiff then went to the offices of the Society, and, upon his producing to them his American diploma, they *relaxed their opposition*, recognising that the practice of American medical men differed from that of the English medical profession." We feel very sure that the Medical Defence Union neither relaxed nor recognised anything on the grounds alleged; and their obvious course, on seeing the diploma which constituted Cunningham's sole claim to practise, would have been to communicate with the Society of Apothecaries, with a view to the commencement of a prosecution. It appeared that Cunningham next became connected with a disreputable "Bad Debt Collecting Association," and also that he continued to advertise and to practise his alleged cure, his so-called consulting-rooms being at the offices of the "Viavi Company." Cunningham next went to the editor of the *Daily Express*, and suggested to him that he should "assist" in making the "discovery" known to the world. The editor agreed to send a medical man to report upon the matter, and did so; but the nature of the report was not divulged, and the only "assistance" given was in the form of the exposure complained of. The time of the Court was occupied, of course, by the usual stories about "cures" and the like, which, equally of course, could not be substantiated; and on the third day of the hearing the jury stopped the case and found for the defendant, Mr. Lawson Walton making the very significant remark that, although while the trial was pending he had felt it to be his duty to do his utmost to ensure that justice was done to the plaintiff's case, after what the jury had said he recognised that his responsibility was at an end. It is probably too sanguine to hope that the defendant will derive any other satisfaction from the trial than the consciousness of having done his duty to society and of having successfully resisted an audacious attempt at robbery.

We cannot but offer to the editor of the *Daily Express* our gratitude for the service which he has rendered to the public, not only in refusing to become the tool of a quack, but also in placing the quack in his true colours before the public. The commencement of a new century affords to other newspapers an excellent opportunity for following so admirable an example.

